# Odontalgia

**Published:** 1851-01

**Authors:** 


*Odontalgia.*—We are requested by Dr. Robinson, to state, that in his work, when speaking of the subdivision of "Odontalgia," he inadvertently omitted giving Dr. S. P. Hullihen, of Wheeling, Va., the credit due to him, as the first to publish the views therein set forth upon the subject.

